# Accessing Autonomic Function Can Early Screen Metabolic Syndrome

**DOI:** 10.1371/journal.pone.0043449

**Published:** 2012-08-20

**Authors:** Kan Sun, Yu Liu, Meng Dai, Mian Li, Zhi Yang, Min Xu, Yu Xu, Jieli Lu, Yuhong Chen, Jianmin Liu, Guang Ning, Yufang Bi

**Affiliations:** 1 Key Laboratory for Endocrine and Metabolic Diseases of Ministry of Health, Rui-Jin Hospital, Shanghai Jiao Tong University School of Medicine, E-Institute of Shanghai Universities, Shanghai, China; 2 Shanghai Clinical Center for Endocrine and Metabolic Diseases, Shanghai Institute of Endocrine and Metabolic Diseases, Department of Endocrinology and Metabolism, Rui-Jin Hospital, Shanghai Jiao Tong University School of Medicine, Shanghai, China; University of Santiago de Compostela School of Medicine - CIMUS, Spain

## Abstract

**Background:**

Clinical diagnosis of the metabolic syndrome is time-consuming and invasive. Convenient instruments that do not require laboratory or physical investigation would be useful in early screening individuals at high risk of metabolic syndrome. Examination of the autonomic function can be taken as a directly reference and screening indicator for predicting metabolic syndrome.

**Methodology and Principal Findings:**

The EZSCAN test, as an efficient and noninvasive technology, can access autonomic function through measuring electrochemical skin conductance. In this study, we used EZSCAN value to evaluate autonomic function and to detect metabolic syndrome in 5,887 participants aged 40 years or older. The EZSCAN test diagnostic accuracy was analyzed by receiver operating characteristic curves. Among the 5,815 participants in the final analysis, 2,541 were diagnosed as metabolic syndrome and the overall prevalence was 43.7%. Prevalence of the metabolic syndrome increased with the elevated EZSCAN risk level (p for trend <0.0001). Moreover, EZSCAN value was associated with an increase in the number of metabolic syndrome components (p for trend <0.0001). Compared with the no risk group (EZSCAN value 0–24), participants at the high risk group (EZSCAN value: 50–100) had a 2.35 fold increased risk of prevalent metabolic syndrome after the multiple adjustments. The area under the curve of the EZSCAN test was 0.62 (95% confidence interval [CI], 0.61–0.64) for predicting metabolic syndrome. The optimal operating point for the EZSCAN value to detect a high risk of prevalent metabolic syndrome was 30 in this study, while the sensitivity and specificity were 71.2% and 46.7%, respectively.

**Conclusions and Significance:**

In conclusion, although less sensitive and accurate when compared with the clinical definition of metabolic syndrome, we found that the EZSCAN test is a good and simple screening technique for early predicting metabolic syndrome.

## Introduction

Metabolic Syndrome is a cluster of metabolic risk factors for cardiovascular disease and type 2 diabetes mellitus. The major components of metabolic syndrome include insulin resistance, excess abdominal fat, dyslipidemia, hypertension and hyperglycemia [1]. Metabolic syndrome is associated with sympathetic activation [2], [3], [4]. Previous study has found that metabolic syndrome is a state of peripheral sympathetic nerve hyperactivity [5]. The higher risk of cardiovascular disease in patients with metabolic syndrome and hypertension could depend partly on the degree of sympathetic hyperactivity. Autonomic dysfunction, therefore, is considered to be one of the most critical pathogenic factors that participated in the development and progression of metabolic syndrome [6].

Autonomic dysfunction is manifested as impaired sympathetic and parasympathetic function, such as hypertension, arrhythmia, and sudomotor dysfunction. Examination of the sudomotor function could be a highly sensitive detection method in distal small-fiber neuropathy and its related autonomic fibers dysfunction [7]. The sudomotor function, therefore, could be taken as a directly reference and screening indicator in predicting metabolic syndrome. Apparently, detection of metabolic syndrome and its relevant autonomic dysfunction in their initial stage could result in appropriate early interventions. However, there are still lack of simple methods and instruments for the early assessment of autonomic dysfunction in those patients.

In this study, we used electrochemical skin conductance measurement to evaluate sudomotor function and to explore whether the method is appropriate for early predicting metabolic syndrome.

## Methods

### Ethics Statement

The study protocol was approved by the Institutional Review Board of the Rui-Jin Hospital affiliated to Shanghai Jiao-Tong University School of Medicine and was in accordance with the principle of the Helsinki Declaration II. Written informed consent was obtained from each participant before data collection.

### Study Population

We performed a population based cross-sectional study in a community of Jiading District, Shanghai, China from March to August 2010. Residents who aged 40 years and older were invited to participate by examination notice and home visits. Totally, there were 10,375 subjects took part in the survey. Among those participants, 6,000 subjects during March to June were attended the EZSCAN test. There were 113 subjects not appropriate for the sudomotor function test for the following conditions: alcohol consumption before testing; amputation of arm or leg; electrical implantable device, such as pacemaker and defibrillator; sensitivity to nickel or any other electrodes; reluctant to accept and cooperate with the test. Individuals who had complete information about age, sex, blood pressure, waist circumference, body mass index (BMI), fasting plasma glucose (FPG), serum insulin and lipid profiles were eligible to the analysis. Therefore, we further excluded participants have incomplete information of metabolic syndrome (n = 72). Eventually, a total of 5,815 individuals (2,339 men and 3,476 women) were included in the current analysis.

**Table 1:** Characteristics of the study population.

	EZSCAN (0–24)	EZSCAN (25–49)	EZSCAN (50–100)	p for trend	p value$
n	697	3069	2049		
Age (years)	51.0±7.8	56.3±8.3*	63.6±10.6*	<0.0001	<0.0001
Male (n, %)	293 (42.0)	1283 (41.8)	763 (37.2)	0.0020	0.0027
BMI (kg/m^2^)	23.5±2.9	24.9±3.0*	25.9±3.6*	<0.0001	<0.0001
Waist circumference(cm)	78.9±8.2	81.9±8.4*	85.0±9.7*	<0.0001	<0.0001
SBP (mmHg)	124.1±15.7	141.7±18.2*	146.0±20.5*	<0.0001	<0.0001
DBP (mmHg)	77.3±9.2	84.2±10.1*	82.6±10.6*	<0.0001	<0.0001
TG (mmol/L)	1.15 (0.83–1.63)	1.33 (0.94–1.91)*	1.48 (1.07–2.06)*	<0.0001	<0.0001
TC (mmol/L)	5.12±0.96	5.34±1.04*	5.38±1.03*	<0.0001	<0.0001
HDL-C (mmol/L)	1.36±0.34	1.34±0.32#	1.31±0.32*	<0.0001	0.0004
LDL-C (mmol/L)	2.98±0.82	3.15±0.85*	3.20±0.89*	<0.0001	<0.0001
FPG (mmol/L)	4.99 (5.59–5.42)	5.15 (4.78–5.67)*	5.32 (4.85–6.21)*	<0.0001	<0.0001
Fasting insulin (µIU/ml)	5.30(3.40–7.40)	6.20 (3.92–9.00)*	7.90 (5.20–11.60)*	<0.0001	<0.0001
HOMA-IR	1.17 (0.74–1.68)	1.45 (0.90–2.15)*	1.92 (1.20–3.10)*	<0.0001	<0.0001
Current smoking, (n, %)	182 (27.0)	727 (24.3)	347 (17.5)	<0.0001	<0.0001
Current drinking, (n, %)	74 (11.0)	371 (12.4)	179 (9.0)	0.0084	0.0007

Data were means ± SD or medians (interquartile ranges) for skewed variables or numbers (proportions) for categorical variables and p values were calculated for the linear regression analysis tests across the three groups.

BMI, body mass index; SBP, systolic blood pressure; DBP, diastolic blood pressure; TG, triglycerides; TC, total cholesterol; HDL-C, high-density lipoprotein cholesterol; LDL-C, low-density lipoprotein cholesterol; FPG, fasting plasma glucose; HOMA-IR, homeostasis model assessment of insulin resistance.

* p<0.001 compared with EZSCAN (0–24) group.

# p<0.05 compared with EZSCAN (0–24) group.

$ p values were for the ANOVA or χ^2^ analyses across the three groups.

### Data Collection

A standard questionnaire was administered to collect information on health status, history of chronic diseases, medications and lifestyle risk factors. The current stage definition of smoker and drinker were subjects who smoked cigarettes or consumed alcohol regularly in the past 6 months. Anthropometric measurements were performed by trained and certified clinical staff members by use of standard protocols and automated electronic device. Three times consecutive blood pressure measurements by the same observer with 1 minute intervals were obtained in all subjects in a sitting position after 5 minutes of rest. The average of three measurements of blood pressure was used for data analysis. Height and weight were recorded to the nearest 0.1 cm and 0.1 kg while participants were wearing light indoor clothing without shoes. BMI was calculated as weight divided by squared height (kg/m^2^). Waist circumference was measured to the nearest 0.1 cm at the umbilical level with participant in standing position.

After at least 10 hours of overnight fasting, venous blood samples were collected for the measurements of plasma glucose (Modular P800, Roche, Basel, Switzerland). Measurement of fasting serum insulin, triglycerides (TG), total cholesterol (TC), high-density lipoprotein cholesterol (HDL-C) and low-density lipoprotein cholesterol (LDL-C) used an auto-analyzer (Modular E170, Roche, Basel, Switzerland). The insulin resistance index (homeostasis model assessment of insulin resistance, HOMA-IR) was calculated as fasting insulin (µIU/ml) × fasting glucose (mmol/L)/22.5 [8].

**Table 2:** Pearson’s correlation and multiple regression analysis of risk factors associated with EZSCAN value.

	r	p value	Standardized β	p value
BMI (kg/m^2^)	0.22	<0.0001	0.27	<0.0001
Waist circumference (cm)	0.21	<0.0001	0.24	<0.0001
SBP (mmHg)	0.26	<0.0001	0.11	<0.0001
DBP (mmHg)	0.03	<0.0001	0.12	<0.0001
TG (mmol/L)	0.10	<0.0001	0.12	<0.0001
TC (mmol/L)	0.06	<0.0001	0.01	0.56
HDL-C (mmol/L)	−0.04	0.0002	−0.12	<0.0001
LDL-C (mmol/L)	0.06	<0.0001	0.02	0.29
FPG (mmol/L)	0.17	<0.0001	0.16	<0.0001
Fasting insulin (µIU/ml)	0.18	<0.0001	0.24	<0.0001
HOMA-IR	0.21	<0.0001	0.26	<0.0001

Multiple regression analysis is adjusted for age and sex.

r, correlation coefficient; β, regression coefficient.

BMI, body mass index; SBP, systolic blood pressure; DBP, diastolic blood pressure; TG, triglycerides; TC, total cholesterol; HDL-C, high-density lipoprotein cholesterol; LDL-C, low-density lipoprotein cholesterol; FPG, fasting plasma glucose; HOMA-IR, homeostasis model assessment of insulin resistance.

### Electrochemical Skin Conductance Measurement

EZSCAN® (Impeto Medical, Paris, France), as a noninvasive, highly accuracy tool for electrochemical skin conductance measurement, has many advantages in the assessment of sudomotor function and is available for both scientific research and clinical practice. Previous studies have shown that through measuring sudomotor function, the EZSCAN test can be used to screen diabetes [9] and access autonomic neuropathy in diabetic patients [10].

Through carrying out a technique call reverse iontophoresis, the EZSCAN test can measure sweat chloride concentrations and give an accurate assessment of eccrine glands and its sympathetic innervations [11], [12]. During the test, six nickel (Ni) electrodes are settled on hands, feet and forehead where the areas of skin are rich in sweat glands. Every two sets of the electrodes are used as an anode and a cathode by turns. Then a direct-current incremental voltage ≥4 V is applied to the Ni electrodes. When the extracted sweat encounters the Ni electrode, a current that proportional to the chloride concentration of the sweat has been created. The electrochemical skin conductance of the sweat can be measure according to the ratio between current generated and the constant direct-current stimulus. Based on the electrochemical skin conductance of hands, feet and forehead, the EZSCAN value, which range from 0 to 100, is calculated with a well-designed algorithm and therefore, can give a time-saved and easy-interpreted result of sudomotor function. Measurement of EZSCAN value is then graphically displayed on a standard personal computer, which is available for an immediate explanation to the participants. Higher readings of EZSCAN value indicated higher risk of any neurological or metabolic abnormalities.

According to the instructions of the EZSCAN, as which recommended by the Impeto Medical Company, small fiber neuropathy and cardio-metabolic risk classified by the EZSCAN value is defined as the following three level: No risk group (EZSCAN value: 0–24); Moderate risk group (EZSCAN value: 25–49); High risk group (EZSCAN value: 50–100).

**Figure 1:**
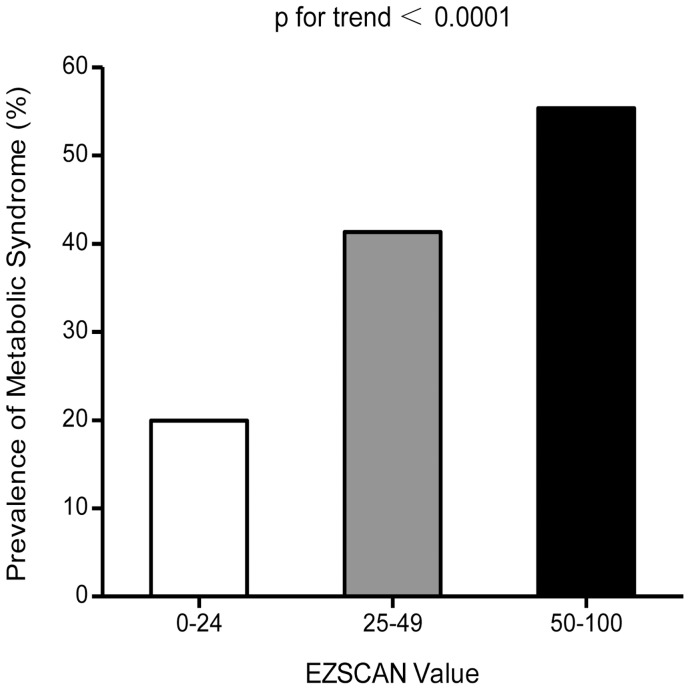
Prevalence of metabolic syndrome in different risk level of EZSCAN value. 0–24 (n = 139); 25–49 (n = 1268); 50–100 (n = 1134).

**Figure 2:**
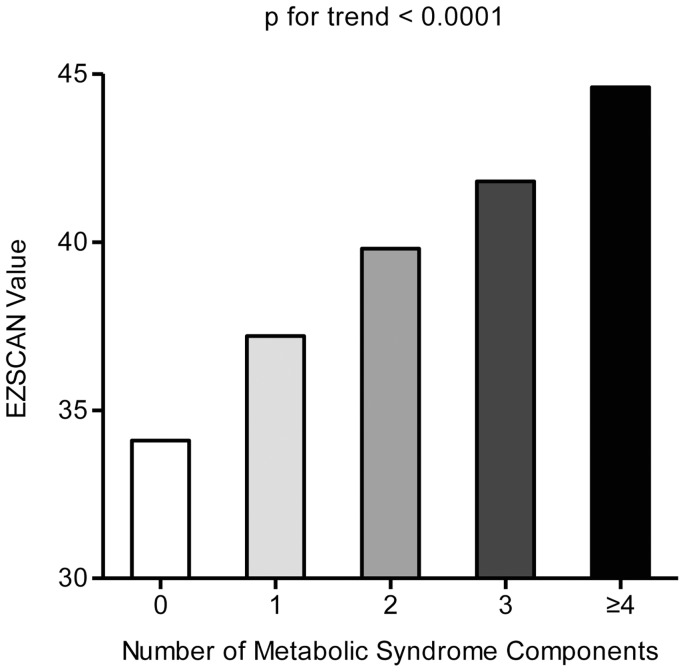
EZSCAN value in different number of metabolic syndrome components. 0, n = 590; 1, n = 1236; 2, n = 1448; 3, n = 1310; ≥4, n = 1231.

### Definition of Metabolic Syndrome and Diabetes

Metabolic syndrome is defined according to the harmonized definition of a joint interim statement of the following organizations: International Diabetes Federation Task Force on Epidemiology and Prevention; National Heart, Lung, and Blood Institute; American Heart Association; World Heart Federation; International Atherosclerosis Society; and International Association for the Study of Obesity [13]. Metabolic syndrome was diagnosed as the presence of three or more of the following abnormal factors: 1. Elevated triglycerides: serum TG concentration of 1.7 mmol/L or greater; 2. Reduced HDL-C: HDL-C concentration of less than 1.0 mmol/L in men or less than 1.3 mmol/L in women; 3. Elevated blood pressure: blood pressure of 130/85 mmHg or greater; 4. Elevated fasting glucose: FPG concentration of greater than 5.6 mmol/L or drug treatment of elevated glucose; 5. Elevated waist circumference: waist circumference equal to or greater than 85 cm in men and 80 cm in women. Cutoff point for waist circumference was based on recommended thresholds for Chinese population. Diabetes was diagnosed according to the 1999 World Health Organization diagnostic criteria.

### Statistical Analysis

Statistical analysis was performed using SAS version 9.2 (SAS Institute Inc, Cary, NC, USA). Continuous variables were presented as mean ± standard deviation (SD) except for skewed variables, which were presented as medians (interquartile ranges). Categorical variables were expressed as numbers (proportions). FPG, TG, and HOMA-IR were logarithmically transformed before analysis due to the non-normal distribution. We used linear regression analysis to test the trend of the demographic and clinical characteristics across the elevated EZSCAN risk level. Differences among groups were tested by one-way ANOVA and *post hoc* comparisons were performed by using Bonferroni test. Comparisons of proportions were performed with the χ^2^ test. Pearson’s correlation and multivariate linear regression models were performed to evaluate the associations between EZSCAN value and BMI, waist circumference, systolic blood pressure (SBP), diastolic blood pressure (DBP), TG, TC, HDL-C, LDL-C, FPG and HOMA-IR. The unadjusted and multivariate adjusted logistic regression analyses were used to investigate the associations of the elevated risk with metabolic syndrome. In model 1, no covariate was adjusted. Model 2 was adjusted for age, sex, and BMI. Model 3 was further adjusted for current smoking, drinking status and antidiabetic medication. Odds ratios (ORs) and the corresponding 95% confidence intervals (95% CI) were calculated for an increase of each risk level in the three different models. The receiver operating characteristics curve was used to examine the diagnostic performance of EZSCAN value for predicting participants with metabolic syndrome [14]. In order to obtain a better assessment of the prediction power of the EZSCAN test, we used the optimal operating point with setting the minimum sensitivity of 70% for which we do not want sensitivity to fall below [15]. The statistical tests were two-sided, and a p value <0.05 was considered statistically significant.

**Table 3:** The risk of prevalent metabolic syndrome according to risk level of EZSCAN value.

		EZSCAN (0–24)	EZSCAN (25–49)	EZSCAN (50–100)	p for trend
Metabolic syndrome	Model1	1	2.83 (2.32–3.45)	4.98 (4.05–6.11)	<0.0001
	Model2	1	1.95 (1.56–2.43)	2.58 (2.02–3.28)	<0.0001
	Model3	1	1.86 (1.48–2.33)	2.35 (1.83–3.01)	<0.0001

Data are odds ratios (95% CI) compared with EZSCAN (0–24) group. Participants without metabolic syndrome are defined as 0 and with metabolic syndrome as 1.

Model 1 is unadjusted.

Model 2 is adjusted for age, sex, BMI.

Model 3 is adjusted for age, sex, BMI, current smoking, drinking status and antidiabetic medication.

## Results

According to cutoff points of EZSCAN risk level mention above, all of the 5,815 individuals were divided into three groups. Table 1 displayed the clinical characteristics of the participants in the present study. As shown in Table 1, increasing EZSCAN risk level was associated with increasing of age, BMI, waist circumference, SBP, TG, TC, LDL-C, FPG, HOMA-IR, and decreasing of HDL-C.

Pearson’s correlation analysis revealed that BMI, waist circumference, SBP, DBP, TG, TC, LDL-C, FPG, and HOMA-IR were significantly correlated with EZSCAN value (Table 2). By performing multivariate linear regression analysis and further adjusted for age and sex, we found that EZSCAN value was positively correlated with BMI, waist circumference, SBP, DBP, TG, FPG, HOMA-IR, and was inversely correlated with HDL-C.

**Figure 3:**
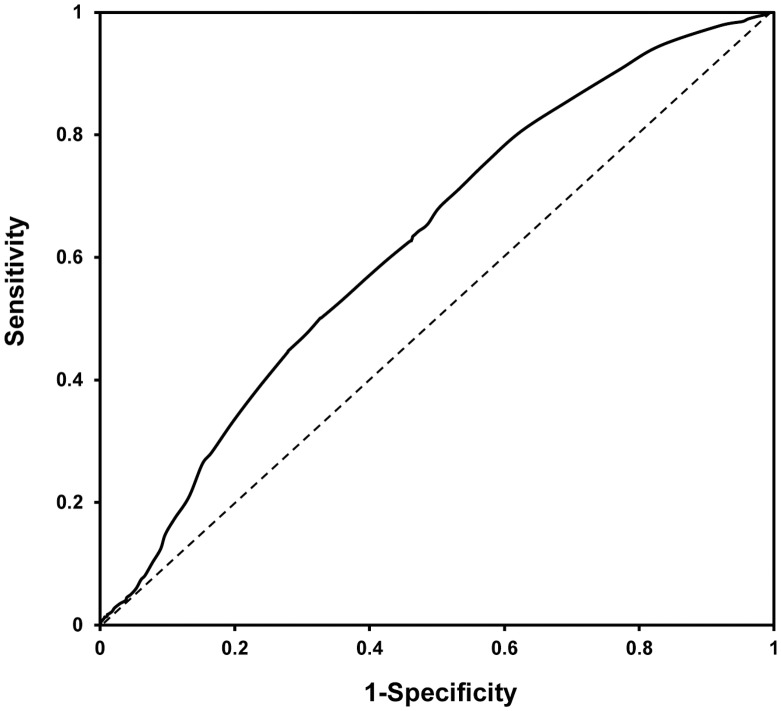
Receiver operating characteristics curve of EZSCAN value for predicting metabolic syndrome. Area under curve: 0.62 (95% CI 0.61–0.64).

**Table 4:** Sensitivity, specificity, positive predictive value, negative predictive value, positive likelihood ratio, and negative likelihood ratio for predicting metabolic syndrome with the EZSCAN test.

	n	Sensitivity	Specificity	Positivepredictive value	Negativepredictive value	Positivelikelihood ratio	Negative likelihood ratio
EZSCAN ≥25	5118	94.5%	17.0%	46.9%	80.0%	1.1	0.3
EZSCAN ≥30	3554	71.2%	46.7%	50.9%	67.7%	1.3	0.6
EZSCAN ≥50	2049	44.6%	72.1%	55.3%	62.6%	1.6	0.8

Among the 5,815 participants, 2,541 were defined as having metabolic syndrome and the overall prevalence was 43.7%. Overall, 1,008 had type 2 diabetes (545 with a previously diagnosis and 463 with a new diagnosis) and 403 of them were on antidiabetic treatment before the baseline survey. The prevalence of the metabolic syndrome increased with EZSCAN risk level, with 19.9% in EZSCAN value 0–24 group, 41.3% in EZSCAN value 25–49 group and 55.3% in EZSCAN value 50–100 group. (Figure 1, p for trend <0.0001). To assess the internal consistency of our observations, we also examined the direct relations of the number of metabolic syndrome components with the EZSCAN value. Since only 315 subjects had all the five components of metabolic syndrome, we performed analysis by combining subjects who had equal to or greater than four components together. As shown in Figure 2, the EZSCAN value further increased with the numbers of metabolic syndrome components (p for trend <0.0001).

Compared with the EZSCAN value at no risk group, univariate logistic regression analysis showed that subjects in the moderate risk group and the high risk group, respectively, have a significant correlation with increased odds of the prevalence of metabolic syndrome. After further adjusted for age, sex, BMI, current smoking, drinking status and antidiabetic medication, the ORs of having the metabolic syndrome for increasing EZSCAN risk level were 1.00 (reference), 1.86 (95% CI 1.48–2.33) and 2.35 (95% CI 1.83–3.01), respectively (Table 3, p for trend <0.0001).

As shown in Figure 3, the receiver operating characteristics curve represented the diagnostic accuracy of EZSCAN value for predicting metabolic syndrome. The area under the curve was 0.62 (95% CI 0.61–0.64) in the study. A threshold of 30 of the EZSCAN value was selected as the optimal operating point to detect metabolic syndrome in this study. When excluding individuals with diabetes, the EZSCAN test became less effective in early predicting metabolic syndrome and the area under the curve turned out to be 0.59 (95% CI 0.57–0.60). Moreover, in order to obtain a better assessment and manifestation of metabolic risks, the sensitivity, specificity, positive predictive value, negative predictive value, positive likelihood ratio, and negative likelihood ratio for identifying metabolic syndrome at thresholds of EZSCAN value 25, 30 and 50 were shown in Table 4. Using thresholds of 25, 30 and 50 of the EZSCAN value, respectively, the sensitivity were 94.5%, 71.2% and 44.6%, while the specificity were 17.0%, 46.7% and 72.1%.

## Discussion

In the present study, we found that the EZSCAN test, as an innovative tool for the assessment of autonomic dysfunction, can be measured easily and quickly to detect metabolic syndrome. To our current knowledge, this is the largest population-based study to explore the association of sudomotor function with metabolic syndrome.

The metabolic syndrome was becoming increasingly common in China. Age standardized prevalence of metabolic syndrome in Chinese populations was 9.8% in men and 17.8% in women in the year 2001 [16]. Diabetes as an important component of metabolic syndrome, was 9.7% in Chinese populations in the year 2008 [17]. Detection and diagnose of metabolic syndrome in the early stage will definitely extend people’s life expectancy and decrease the burden of the government. As the only regulatory system of the sweat glands, the sympathetic nervous system plays a central role in the body metabolic balance [18], [19]. An early marker of small fiber neuropathy is the abnormal sweat response to people who have sympathetic dysfunction [20]. Autonomic status, therefore, can be assessed by sudomotor function [21], which can also be an efficient screening tool for autonomic related diseases [22].

Accessing sudomotor dysfunction by the EZSCAN test was first validated in cystic fibrosis patients, as compared with control subjects, a diagnostic specificity of 100% and a sensitivity of 93% were provided [23]. After this, a French study showed that the EZSCAN test can also provide a good sensitivity of 75% and specificity of 100% for assessing sudomotor dysfunction in people with diabetes [10]. Similarly, Sheng et al. [24] found that in Chinese people, the EZSCAN test has a well performance in diabetes detection and diagnosis, the sensitivity and specificity were 85% and 64% in their study. In addition, a recent research found that the EZSCAN test could be used as an efficient test in predicting high-risk subjects for diabetic nephropathy [25]. Ramachandran et al. [9] even gave a conclusion that the EZSCAN test could potentially replace FPG as a screening method for the early assessment of abnormal glucose metabolism. In the same research, they also pointed out that the EZSCAN test performed good sensitivity in predicting normal glucose tolerance individuals with metabolic syndrome. In the present study, we found that many individuals with metabolic syndrome also be excluded (818 individuals in the present study) when excluding individuals with diabetes. As dysglycemia is a component of metabolic syndrome, including people with impaired glucose regulation and diabetes could better reflect the performance of the EZSCAN test in predicting the metabolic syndrome.

The threshold that indicated the most appropriate trade-off between sensitivity and specificity, is usually selected by the closest point to the left upper corner of the receiver operating characteristics curve, especially for the diagnostic purpose. However, the EZSCAN test, as an easily administered and noninvasive implement, is more appropriated for early detection of metabolic disorders during clinical trials and epidemiologic studies. A missed diagnosis of metabolic syndrome could be harmful for both treatment and prognosis of the disease. In this case, it is more crucial to have a high probability of the EZSCAN test for the prediction of a responder as compared to the probability of a non-responder. Therefore, we analysis the optimal operating point for the screening function of the EZSCAN test [15]. With a pre-assigned threshold of sensitivity for predicting a true-responder, greater than 70% in the present study, 30 of the EZSCAN value was thought to have a more appropriate predicting effect for metabolic syndrome, of which the sensitivity and specificity were 71.2% and 46.7%, respectively.

Early assessment of the risk of metabolic syndrome is extremely important. However, techniques for its early stage detection were rare. Nevertheless, the technical improvements have allowed a more precise assessment of autonomic function, by which can be used as a novel method for early detection of metabolic syndrome. In the present study, we found that the EZSCAN test appears to have many advantages in the assessment of sudomotor function and its related metabolic disorders. Firstly, the whole test is almost automatic and the result is displayed in two minutes. Secondly, neither special preparation for the participants nor medical training for the operator is necessary. What’s more, the test is available and more acceptable for children among who have the early onset of metabolic syndrome and many metabolic abnormalities [26], [27]. No technique available so far can be taken as the best method for assessing autonomic function with which the others might be compared. Quantitative Sudomotor Axon Reflex Test (QSART) is one of the most common test in assessing autonomic nervous system disorders, peripheral neuropathies, and some types of pain indispositions. The highly professional expertise requirement, long time-duration, and the low reproducibility [28], however, are limited its application in large-scale epidemiological studies and regular follow-up. Therefore, the EZSCAN test is perfectly suited for general practitioners, pharmacists, and neurologists for routine application.

There are still several limitations needed to be noted. First, compared with the clinical definition of metabolic syndrome, the EZSCAN test for predicting metabolic syndrome has certain weakness. The metabolic syndrome includes a number of essential abnormalities that occur together more often than by chance alone. Only by assessment of the autonomic function, the EZSCAN test could be relatively less sensitive and accurate. However, accomplished by several anthropometric measurements and fasting blood test, clinical diagnose of the metabolic syndrome is invasive and time-consuming. In comparison, the EZSCAN test is more convenient to execute and provides immediate results in two minutes, without any need for patient preparation or blood drawing. Accordingly, these advantages could make it more acceptable and feasible in screening and follow-up population at high-risk of metabolic syndrome. Second, as the EZSCAN test is more suitable for screening purpose and not for diagnosis, it is recommend that other factors, such as blood pressure, lipid profiles, and BMI should be taken into account for a better evaluation of metabolic syndrome. Third, the study was done in a Chinese population aged 40 years or older, future studies should evaluate performance of the EZSCAN test of subjects in different ages and races. Supported by our findings, further longitudinal studies to assess the predict power of measurement of autonomic function in metabolic syndrome are also demanded.

In conclusion, although has certain weakness when compared with the current definition of metabolic syndrome, we found that the EZSCAN test is a good and simple method for early screening metabolic syndrome.
